# Closing the barrier between disease and health outcomes in Africa through research and capacity development

**DOI:** 10.1080/16549716.2018.1425597

**Published:** 2018-01-26

**Authors:** Beverley Kramer, Elena Libhaber

**Affiliations:** ^a^ Health Sciences Research Office, University of the Witwatersrand, Johannesburg, South Africa; ^b^ School of Anatomical Sciences, Faculty of Health Sciences, University of the Witwatersrand, Johannesburg, South Africa

**Keywords:** Research, research management, research infrastructure, capacity development, training

## Abstract

**Background**: While the burden of disease in Africa is high, health research emanating from the continent is low. Building human capacity and research infrastructure to close the gap between research and disease is thus of great imporatance.

**Objective**: In order to improve research outputs and postgraduate training in the Faculty of Health Sciences, University of the Witwatersrand, the Health Sciences Research Office put in place a series of strategic initiatives over time.

**Methods**: A range of strategic activities, for both postgraduate students and academic staff, were developed in parallel and sequentially over a period of approximately nine years (2008–2016). The latter years were a time of consolidation of the programmes. Outcomes of these activities were ‘measured’ by increases in publications, decreases in time to graduation and enrichment of the research environment.

**Results**: A doubling of research publications and an increase in citations occurred over the period under review. In addition, there was a decrease in the time postgraduate students took to graduate.

**Conclusions**: A varied, but structured research management plan may be of value in African and other developing health sciences institutions to enable the increase in research outputs and capacity development, desperately needed to close the barrier between disease and health.

## Background

While the Global South carries the greatest burden of the world’s health problems, capacity to support health research in developing countries remains one of the world’s unmet challenges [1,2]. Fonn [3] maintains that research which is ‘conceptualised, conducted, analysed and published’ by Africans is central to meeting the health needs of Africa. While progress in capacity development in Africa has occurred in certain areas – for example, epidemiological research and ethics research [4] – there is widely acknowledged agreement of the pressing need to develop and nurture scientists in Africa [5]. In particular, the nurturing of health scientists is extremely important [4,6–8] in order to prevent disease and premature deaths, as well as to improve equity.

However, health and health systems across Africa will not improve, unless strategic research platforms are set up and unless capacity development is consciously targeted. Key requirements for the strategic improvement of health research were identified by the members of the Initiative to Strengthen Health Research Capacity in Africa (ISHReCA) and included improving the research environment, support for individuals and support for institutions [9]. In addition, at a conference on ‘Mobilisation and Training for the Improvement of Research and Innovation Management in the Central Africa Region’ in 2010, delegates identified key learning points for implementation at their institutions [10]. Health research is vital for improving health in Africa [9].

Due to the quadruple burden of disease (HIV, TB, non-communicable diseases and violence/trauma) in South Africa [11], the academic and clinical staff at institutions deal with excessively high patient numbers and clinical workloads. While the Royal College of Physicians (UK) has stated that the greatest obstacle to doctors carrying out research is time [12], this problem is multiplied considerably in developing countries. It is thus imperative to provide a supportive environment for clinicians to enable the undertaking of research.

The importance of building capacity among health workers from disease-burdened and developing countries has been recognised by many researchers/strategists [2,9,13–21]. Nchinda [2] described steps taken to build capacity with special emphasis on the identification and training of trainees, as well as mechanisms for research sustainability. Most importantly, he proposed that ‘[b]uilding research capacity in the South is too important to be left to chance’ [2]. The CARTA programme (see below) has been one such programme which has focussed on training health sciences researchers in Africa for the future and has ensured that the training of these young scientists would make them globally competitive [7,21].

A number of published works describe mechanisms to strengthen capacity development, or to improve outcomes of health research in Africa [9,17,20,22–26], yet little is known about the results of these programmes. In addition, measuring the success of these strategies is not easy [9]. In this article, we describe initiatives to improve research engagement and capacity development and provide some of the outcomes. While the outcomes are substantial, it is difficult to always link these directly to the initiatives. We have however attempted to evaluate certain aspects of these initiatives [see e.g. 27–30]. We acknowledge that there are other programmes and interventions in the WITS FHS, such as the CARTA programme, the input of the Human Research Ethic Committee (Medical), the administration from the Postgraduate Office, and individual research endeavours and collaborations, which would also have contributed substantially to these outcomes.

While training programmes, seed grants, mentorship programmes and partnerships have been established in Africa [6], these interventions have failed to ‘create a critical mass of well-trained and networked researchers across the continent [or] to increase research productivity’ [7].

### Aim of the study

The present study describes a research management programme and processes which were developed over approximately nine years by the Health Sciences Research Office (HSRO) in the Faculty of Health Sciences, University of the Witwatersrand, Johannesburg, South Africa and which we intended would contribute to improving research outputs, time to completion of postgraduate students and the general research environment.

## Methods

Permission to utilise Health Sciences Research Office data was provided by the Human Ethics Research Committee (Medical) of the University of the Witwatersrand (M170772). This study is a descriptive study which utilised information and data collated over nine years from the operationalisation of strategic initiatives.

At the beginning of 2008, in the University of the Witwatersrand Faculty of Health Sciences (Wits FHS) there was little research support for postgraduate students and staff, with low publication outputs (414 publications per annum) and lengthy times to graduation for postgraduate students (throughput). There was no specific allocation of time for research in the day-to-day activities of academic staff, and researchers in the Faculty of Health Sciences at Wits were expected to make time to do research.

### Initiatives introduced since 2008

**Figure 1.**
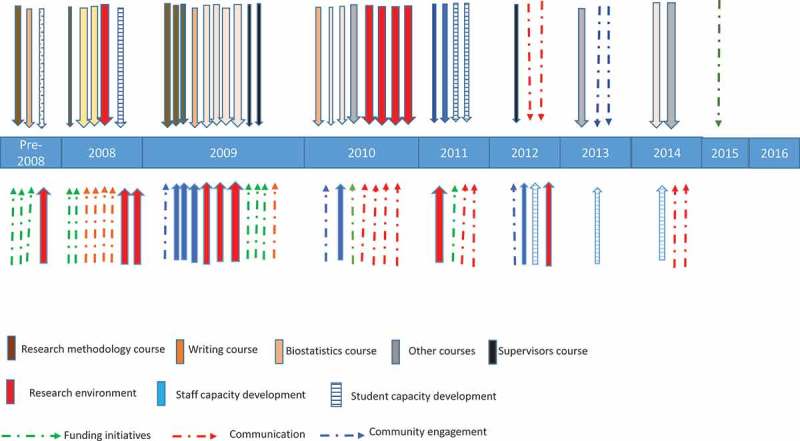
Timeline showing strategic research initiatives which were introduced in the Wits Faculty of Health Sciences over the period 2008–2016. Note that the pre-2008 situation is included for comparison. Each arrow illustrates an unitive that was introduced (One arrow, one initiative).  This figure is fully explained by the contents of supplementary Table 1.

A plethora of activities – that is, over 70 courses, incentives or other initiatives such as writing retreats, funding opportunities, forums, workshop, and intensive Faculty-wide communication – were developed sequentially and in parallel. These were used to support researchers and postgraduate students and are illustrated in Figure 1 (and supplementary Table 1). These initiatives were initially small, but enlarged as knowledge of them spread through the Faculty.

### Training courses

Implementation of courses was aimed at training postgraduate students and emergent researchers through all stages of research from protocol development to publishing an article. Courses in both quantitative and qualitative methodology, data management, basic and advanced biostatistics [27], grant application writing and scientific writing [28] were introduced. In addition, as we progressed, courses in supervision, biostatistics for supervisors and ‘how to mark a thesis/dissertion’ were added for staff members (Figure 1 and supplementry Table 1). Research methodology and biostatistics courses, which were first initiated for postgraduate students and staff, were then also introduced at undergraduate student level.

### Financial assistance/incentives

While there have been substantial financial difficulties in South Africa and particularly in the health sector over the past number of years [31], sufficient resources were gleaned to enable the research and postgraduate endeavour to expand. In 2008, the finances available for postgraduate and staff support through the HSRO in relation to research was approximately R1 million (USD ± 80,000). Thereafter this funding increased, but particularly so in the years 2014 and 2015 (R 3.5 milion to R4 million; ±USD 269,000–308,000). The increased funding was obtained from the Faculty. In 2016, however, funding decreased to R1.7 million (±USD 131,000). Some of the initiatives were started with minimal financial input, but as more funding was obtained, some initiatives were expanded.

While individual grants for research projects for junior staff and postgraduate students were already in place in the Faculty in 2008, a variety of other incentives – such as financial incentives for published articles by postdoctoral Fellows, short-term financed periods for the completion and submission of journal articles by postgraduate students, and seed funding for new, exceptional research projects – were introduced.

### Carnegie academic medicine clinician scientist programme

The Wits Faculty of Health Sciences initiated a programme to train clinician scientists in 2011 [30]. This programme, funded by the Carnegie Corporation of New York, is relatively small (four Fellows per annum are trained due to financial constraints). The programme provides ring-fenced research time for clinicians to undertake a PhD.

### CARTA

This important initiative was established by the Consortium for Advanced Research Training in Africa (CARTA) in 2009. It comprises institutions in Kenya, South Africa (Wits), Tanzania, Uganda, Malawi, Nigeria and Rwanda with key northern partners. Members of staff of the Wits School of Public Health have been intimately involved with setting up and running this programme. The aim of CARTA is to strengthen research infrastructure and train future academics in Africa through the training of PhD candidates and their supervisors. This would enable the researchers to work in multidisciplinary health research environments in the future [7,21]. CARTA funds the training of these individuals and provides workshops for administrators of the contributing institutions. At Wits, students have obtained their PhDs as a result of this programme, and administrative staff – for example, the Faculty Registrar and Assistant Dean amongst others – have been invited to workshops.

### Alumni diaspora research programme

South Africa suffered for many years under the yoke of apartheid, and thus a significant number of clinicians and researchers emigrated. As health research has become a global activity and in order to reignite the interest of Wits alumni in research in South Africa, we initiated an Alumni-Diaspora Research programme [29] in 2010. This programme, which builds research collaborations and networks between the alumnus/a’ institution and the Wits FHS, was enhanced by a grant from the Carnegie Corporation of New York in 2014. This grant allowed expansion of the programme.

### The research environment and mentorship

In order to attempt to develop a fertile and vibrant research environment, we initiated the following: writing retreats for academics and postgraduate students, a postdoctoral forum, an emergent researchers forum, various financial incentives, research lectures (which also allowed interaction with the community) and other forms of engagement (Figure 1; supplementary Table 1), as it was believed that these could be conducive to individual and group development.

In addition, over time, the following were initiated: Postgraduate Hubs (24-hour computer and lounge facilities) at two sites; a Postgraduate Exhibition (South African grant funding agencies, Research Entities offering projects, equipment and consumable suppliers, postgraduate services) was added to the Faculty Research Day; a Research Entity Directors Forum and a Postgraduate Co-ordinator’s Forum were all initiated (Figure 1; supplementary Table 1). These initiatives were employed in an attempt to stimulate communication and bring together researchers from disparate and also geographically widely separated sites in the Faculty.

We believed that an important aspect of doing research, particularly in a large institution, is to provide thanks and recognition for those undertaking research. For this reason a Research Awards Dinner was introduced in 2009, at which the top performing researchers and groups in the Faculty were awarded a certificate of research excellence. In addition, each year the Assistant Dean would nominate some of the top researchers in the field for prestigious awards offered by national funding bodies and other organisations.

In 2009 and 2010, through a grant received by the university, the HSRO was able to set up one-on-one mentorship sessions for some staff. Unfortunately this funding was terminated at the end of 2010. While the HSRO did not have sufficient resources to continue to provide this type of mentorship to all emerging researchers and postdoctoral Fellows in the Faculty, it set up and provided ‘group’ mentorship through two forums. In 2010, an Emergent Researchers Forum and a Postdoctoral Forum were initiated. During these informal forums, items such as how to compose a curriculum vitae (CV), how to develop a CV, reviewing an article through the eyes of an editor, how to communicate research with the media and other areas of interest were discussed.

### Quantitative data on publication output and postgraduate throughput

The number of publications per annum between 2008 and 2016 were obtained by manual counts of publications submitted to the HSRO and also data from the university’s publication ‘collections system’. Wherever possible, these numbers were also compared with data obtained from Pubmed and Scopus.

Data for postgraduate throughputs per annum were received from the university’s Management Information Unit.

## Results

The principle of taking small steps each year, and consolidating and enlarging these over time as additional funding became available or HSRO posts increased, appears to have positively impacted the research outputs and postgraduate throughput of the Wits FHS.

### Courses

The introduction of a variety of courses for the training of postgraduate students and academics may be linked to the improved throughput rates of students between 2008 and 2015. For example, the average time taken to completion of a PhD for full-time students reduced from 4 years in 2008 to 2 years in 2014 and for some master’s degrees (research and course work) from 67 months to 30 months.

Courses in both quantitative and qualitative methodology, data management, basic and advanced biostatistics [27], grant writing, scientific writing [28] and supervision appear to have positively impacted the postgraduate students.

### Finances

The incentives provided to staff and students appear to have made a contribution to the research output in the Faculty as is evidenced by the increase in publications (see below). Individual grants enabled postgraduate students to undertake research. Similarly, start-up incentives and seed funding allowed new staff and emergent researchers to initiate their own research. Many initiatives have now also been translated into practice in the schools of the Faculty; for example, writing retreats and postgraduate dyads

### Carnegie academic medicine programme: clinician scientists programme

The programme, which had its first intake of four Fellows in 2011, has thus far qualified 13 Fellows out of an intake of 20 over the past years. The graduates have been nested back into their clinical domains to initiate clinical research and support capacity development.

### CARTA

The Faculty has benefitted immensely from this programme, not only through the qualification of exceptionally well-trained PhD candidates, but also from the exposure of academic and administrative staff to training. This programme contributes to the increased numbers of publications and numbers of graduate students in the Faculty, and also to the enrichment of the research environment.

### Alumni diaspora research programme

The programme has led to the establishment of a number of ongoing research collaborations (29), joint grant applications, joint research publications, capacity development, and scientific and grant writing courses for postgraduate students and emergent researchers. Collaborating institutions are based in the US, Australia, New Zealand and Europe.

### Research environment and mentoring

The writing retreats led not only to the submission of publications, but also to the development of collegial relationships between disparate academics [28] and in some cases to interdisciplinary collaborations. Lack of time is a major obstacle to writing [28] and thus writing retreats provided some researchers with the opportunity not only to write up their research, but also to form collegial bonds.

Together the writing retreats, the different research forums, the research lecture series and other initiaves resulted in interaction between academics from different disciplines. Through these mechanisms, internal interdisciplinary collaborations were set up and bureaucratic processes, which had a negative impact on research, were identified and brought to the attention of the relevant administrators.

### Outcomes/publication numbers and postgraduate throughputs/graduations

**Figure 2.**
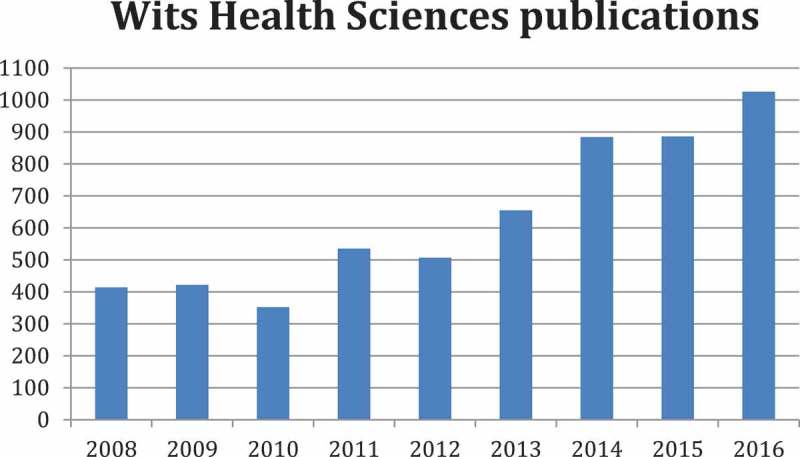
Number of articles per annum published by the Wits Faculty of Health Sciences over the period 2008–2016.

Publication numbers in the Wits FHS more than doubled from around 400 per annum in 2008 to a substantial 1026 per annum in 2016 (Figure 2) and with a considerable increase from 2015 to 2016 alone of 13.5%. The university was also finally recognised within the top 100 institutions at 77th place in the Times Higher Education World University Rankings in the Clinical, Pre-Clinical and Health subject area [32].

**Figure 3.**
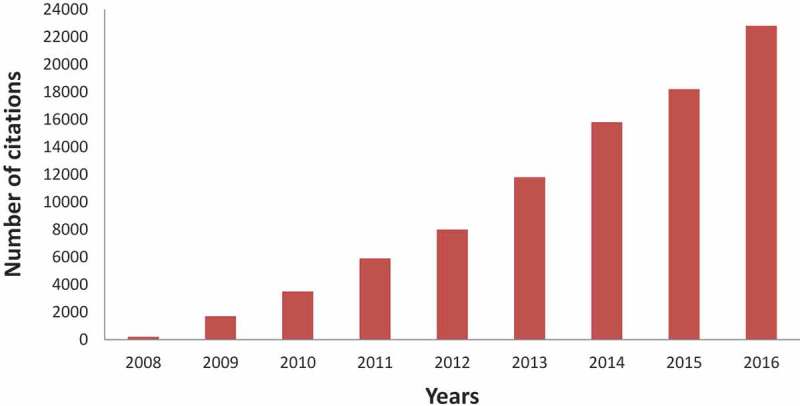
Wits Faculty of Health Sciences citations (derived from Scopus) between 2008 and 2016.

While quantity is important from the institutional perspective, it is not the only metric that should be considered, as the quality of output is equally valued. Eighty percent of the Wits FHS articles are published in ISI-listed (Institute for Scientific Information) international journals, many of these in high impact factor journals. Citation rates of articles emanating from the Wits FHS have markedly increased over the period under review (Figure 3).

### Award for research management

In order for the Faculty to increase its output and improve its support for capacity development of postgraduates and emergent researchers, it was necessary for the Assistant Dean and the Research and Postgraduate Offices to play a much more strategic leadership role. These efforts were formally recognised by the presentation of a prestigious South African Research and Innovation Management Award (SARIMA) to the Health Sciences Research Office in 2015 by SARIMA and the South African National Department of Science and Technology. This award, for ‘Excellence in Research and Innovation Management, celebrates excellence in research and innovation management in Southern Africa. The award acknowledged growth and achievement in the field of research and innovation management as a key enabler of research and innovation’ [33].

## Discussion

Research plays a vital role in closing the barrier between disease and health, particularly in resource-constrained countries. Through research, the mechanisms of disease, diagnostic accuracy, treatment strategy and policy can be advanced. There is little health research capacity in Africa, and thus rebuilding research capacity is urgent. This has been initiated in some fragile states – for example, Somalia [34] – and in other regions (Malawi, Zambia and Zimbabwe) [35]. However, African research capacity has not yet reached the levels of that in high-income countries [36]. Although research areas of excellence already existed in the Wits Faculty of Health Sciences, and some of our researchers have contributed extensively to the international body of knowledge around HIV/AIDS, tuberculosis, malaria and drug delivery [see, for example, 37–43], there was a need to strengthen wider areas of research as well as increase the training and support for emergent researchers and postgraduate students in the Faculty. We believed, like Fonn [3], that it was important to initiate these strategies locally and ensure their sustainability.

As indicated by Whitworth et al. [9], measuring the success of programmes which seek to improve research and capacity development is not easy. Our experience, as described above, has shown that careful strategic implementation of a variety of mechanisms and programmes has benefited the Faculty over time in many ways: the doubling of publications, an increase in citation rates, a decrease in the throughput rate of postgraduate students and an improvement in the research environment and communication. The outcomes of these research strategies, as well as the continued pursuance of excellence by many of the individual researchers and research units, has elevated the institution in relation to local and international standing [32]. While the direct assessment of the outcomes from this strategy is difficult, dissemination of these processes/mechanisms is important for institutions both in this region and in other developing countries.

Two very important lessons were learned during the implementation of the strategy. There are a multitude of agendas in the Faculty [44] as there are in all institutions of higher education, and it thus took strong direction and decision-making to guide the Faculty. In addition, academics cannot be forced to accept change. The strategies and changes introduced by the Health Sciences Research Office did not occur in isolation, but were supported by additional programmes, such as the CARTA programme [7], the Faculty’s research management committees and the individual researchers and research units who were willing to embrace the changes. The power of the collaborative spirit and goodwill of researchers and staff should not be underestimated in setting up research management systems. It was important to engage with all levels of academics to ensure that the majority could see the value in the change or structure that was being set up.

While many research collaborations were already in existence, the Alumni Diaspora programme sought to actively pursue research collaborations with alumni who have an invested interest in the growth of their alma mater [29]. To this end not only were north–south collaborations initiated, but south–south collaborations were seen as equally important. Like Lansang and Olveda [14], we see these collaborations as ‘strategic bridges’ for research and capacity strengthening. Some of the cemented collaborations are those with Vanderbilt Medical School and Johns Hopkins Medical School in the US, the Universities of Queensland and New South Wales in Australia, the University of Otago in New Zealand and the Universities of Northampton and Oxford in the UK, amongst others. This resource is being considered by institutions in fragile states as well [34].

One of the most important issues in capacity development is mentorship. Mentorship, as differentiated from supervision, is defined by Strauss et al. [45] as ‘providing an enabling relationship that facilitates another person’s growth and development’. When funding for individual mentorship could no longer continue, the introduction of ‘group’ mentorships through the Emergent Researchers Forum and also through the Postdoctoral Forum were two initiatives which were put in place to enable some mentorship. Should funding become available, it would be important to introduce more one-on-one mentoring for emergent researchers and postgraduate students in the future, as Harries et al. [46] suggest that ‘hands-on’ mentoring increases the likelihood of course completion and the publishing of papers.

It should be acknowledged that the assessment of the outcomes of our research management plan and association with the initiatives is limited. It may be that one or more of the initiatives produced the desired outcomes. However, these outcomes cannot be ignored. Key requirements for the strategic improvement of health research were identified by the Initiative to Strengthen Health Research Capacity in Africa (ISHReCA) [47] and by the Congress on the ‘Mobilisation and Training for the Improvement of Research and Innovation Management in the Central Africa Region’ [10]. Some of these requirements were attained by the HSRO, such as the building of an enabling research environment; support for individuals, particularly emergent researchers, through both financial and non-financial means; the creation of support systems for the promotion of research; and the provision of an enriched environment for capacity development.

Like Nchinda [2], who provided reasons for the success of research capability strengthening and lessons learned, from our own experiences we include amongst others: training of emergent researchers, linkages with stronger institutions adequate equipment, training in research, protection of time, suitable mentors, communication, and creating an enabling environment. While little is found in the literature regarding the building of a ‘research environment’, our experience has shown that this aspect is as crucial as the tangible aspects of research management. Rutherford [44] proposes the development of a broad range of experiences within the research management arena in order to strengthen knowledge and develop career pathways. The formation of a structured research management system within the Faculty of Health Sciences, with the systematic introduction of courses, workshops, incentives, forums and so on, has enabled accomplishments by the research staff and postgraduate students in the Faculty. The development of a spread of initiatives, like the spokes of a wheel, empowered a comprehensive strategic outcome.

Sustainability of the programme in the future will be linked to ongoing strong leadership and continued financial support. This refers not only to maintaining the infrastructure required for research, but also to the collegiality and collaboration (both internal and external) required to progress. While funding is important, we have shown that it is possible to initiate structures with very limited financial resources (e.g. range of courses, group mentoring, Postgraduate Hub) and although there was a decrease in funding in 2016, the initiatives were sustained.

## Conclusion

While mechanisms do not exist to measure outcomes in the research management arena, the doubling of research publications and the increased citations over the period under review could be indirectly linked to the strategy. Thus, the initiatives utilised by the Wits HSRO research management team may be of value to other institutions for improving the much needed capacity development and health research output required to strengthen health care for the future in Africa and other developing countries.

## Supplementary Material

Supplementary material

## Paper context

Health research is of paramount importance in a developing country which is burdened with disease. Little existed in our institution to support research and capacity development. Strategic initiatives were thus introduced, in parallel and chronologically, over a period of nine years. Publication of health research and the graduation of students were some of the results of these initiatives. These initiatives may be of assistance to institutions in other developing countries facing similar difficulties.
