# Supplementary material for: Closing the barrier between disease and health outcomes in Africa through research and capacity development
Source: Glob Health Action. 2018 Jan 26;11(1):1425597. doi: 10.1080/16549716.2018.1425597 (PMC5795707; doi:10.1080/16549716.2018.1425597)
Supplement: Supplementary material [file ZGHA_A_1425597_SM0381.docx]

|  | **Activity** | **Activities prior to 2008** | **2008** | **2009** | **2010** | **2011** | **2012** | **2013** | **2014** | **2015** | **2016** |
| --- | --- | --- | --- | --- | --- | --- | --- | --- | --- | --- | --- |
| S**taff and Postgraduate Development courses:** | Research methodology | Research methodology | | Research Methodology expanded  Qualitative research methods workshops  One-on-one consultations with individuals on research methodology | Scientific writing course by international experts for staff and postgraduate students |  |  |  |  |  |  |
|  | Scientific writing | Basic scientific writing skills |  | How to write a literature review |  |  |  |  |  |  |  |
|  | Biostatistics |  | Biostatistics basic course  Acquisition of two Statistical programmes | Applied biostatistical methods  Sample size workshop  Data entry and management workshop  Biostistics one-on-one consultations with individuals  Biostistics seminars | Advanced biostatistics course  Statistical packages workshops |  |  |  | Monthly data analysis workshops utilising investigators own data |  |  |
|  | Other courses |  |  |  | Grant writing for staff by international experts |  |  | Redcap workshops | Research integrity |  |  |
|  | Supervisor course and support group | Supervisor support group |  | Supervisor course  How to mark a dissertation/thesis |  |  | Novice supervisors course |  |  |  |  |
| **Community engagement** |  |  |  | Prestigious Research Lecture series | Malaria awareness campaign on radio |  | Cardiovascular Symposium on insights into the management of cardiovascular disease in women | Contribution of research articles to community magazine  External media (newspapers) liaison – to communicate research to community |  |  |  |
| **Capacity development** | **Staff** |  |  | Staff mentoring initiated  Start-up research funding for new staff  Paid time off for staff to complete/degrees/publications | Staff writing retreats | Introduction of a PhD program for Clinician Scientists  Initiation of patient database system for research purposes | Establishment of REDCAp© (research database) in the Faculty with the assistance of Vanderbilt Medical School and the initiation of a Biomedical informatics system |  |  |  |  |
|  | **Students** |  | Incentive for conversion of dissertation /thesis to publication |  |  | Science communication with the media -training for young scientists  Postdoctoral Fellows Symposium | Postgraduate writing dyads to complete dissertation/thesis | Workshop on media engagement and public speaking | Postgraduate writing retreats for dissertation/thesis or publication |  |  |
| **Research Environment** |  | Research Day | Prestigious awards for Postgraduate degrees  Research Day and Postgraduate Expo | Postgraduate Hub initiatedat Medical School  Faculty Research Award dinner  Research co-ordinators forum | Alumni Diaspora Programme initiated  Major collaboration as a result of Alumni Diapora programme  Initiation of Research Entity Forum | Postgraduate Hub at Academic Hospital | Engagement with external funders in order to enlarge Research Day and Postgraduate Expo |  |  |  |  |
|  |  |  |  |  | Postgraduate social event |  |  |  |  |  |  |
|  | **Funding initiatives** | Staff Congress travel  Research equipment (major) contribution  Individual grants | Matched funding towards major equipment  Funding for minor equipment in Faculty | Payment for articles published in high impact or online journals  Postdoctoral Fellows incentive for publishing an article  Pre-paid membership with journal for reduced page charges | Funding support for minor equipment intiated | Postgraduate student Congress travel |  |  |  | Seed funding for staff research projects |  |
| **Communication** |  |  | Production and circulation of grant directory  Research Office Webpage  Research Office Newsletter  Annual Research Office report to Faculty and University | Postgraduate brochure | Initiation of Emergent Researchers Forum  Initiation of Postdoctoral Fellows forum  Postgraduate lunchtime research talks  Digital signage set up, carrying latest research information | Faculty Biennial research report  Research Office Bulletin – important and urgent monthly notifications | Created a postgraduate Facebook page  Initiated a postdoctoral Brochure with information for Fellows and hosts |  | Comprehensive electronic postgraduate information booklet – all information require by postgraduate students and supervisors from admission to graduation contained in book  Portal for offering research projects on the Website |  |  |

Table 1: Parallel and sequential initiation (by year) of the activities introduced by the Health Sciences Research Office for the staff and postgraduate studets of the Faculty
